# Supplementary material for: Statistically defined visual chunks engage object-based attention
Source: Nat Commun. 2021 Jan 11;12:272. doi: 10.1038/s41467-020-20589-z (PMC7801661; doi:10.1038/s41467-020-20589-z)
Supplement: Supplementary file 1 — Supplementary Information [file 41467_2020_20589_MOESM1_ESM.pdf]

# Statistically defined visual chunks engage object-based attention

Gábor Lengyel[1,2]*, Márton Nagy[1,2,3] & József Fiser[1,2]*

[1]Department of Cognitive Science, Central European University, Budapest, Hungary

[2]Center for Cognitive Computation, Central European University, Budapest, Hungary

[3]Department of Cognitive Psychology, Eötvös Loránd University, Budapest, Hungary

SUPPLEMENTARY MATERIALS

*Correspondence to: fiserj@ceu.edu, lengyel.gaabor@gmail.com

## Experiment 1

The median reaction time and mean error rates of the observers for the three types of response in all blocks are shown in Supplementary Fig. 1 for the main (a), replication (b), and in the control (c) experiments. Although the task was difficult and produced relatively high error rates, observers paid attention to the task as indicated by the longer search times in each experiment in trials with only one target letter *T*. In the following, *R1* refers to trials with two target letter *T*s appearing vertically arranged on top of each other, *R2* refers to trials with two target letter *T*s appearing horizontally arranged next to each other, and *R3* denotes trials with only one target appearing.

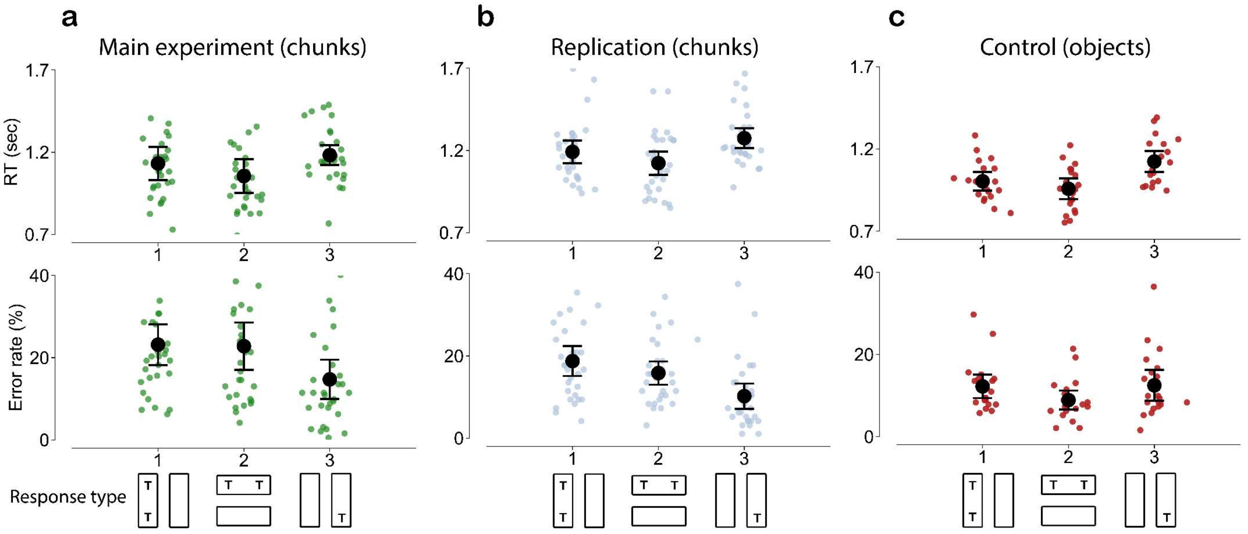


**Supplementary Figure 1.** Median reaction times (**top**) and mean error rates (**bottom**) for the three response types in the main (Experiment 1a, column **a**), in the replication (Experiment 1b, column **b**), and in the control tests (Experiment 1c, column **c**). The response types are shown on the x axis: (1) two target letter *T*s appearing vertically arranged on top of each other, (2) two target letter *T*s appearing horizontally arranged next to each other, and (3) only one target appearing. Error bars in all plots show 95% confidence intervals of the mean. Colored dots represent the mean error rates or median reaction time of the observers. n=30 in Exp. 1a (**a**), n=30 in Exp. 1b (**b**), and n=20 in Exp. 1c (**c**). Source data are provided in the Source Data file (Supplementary Figure 1 worksheet tab in Source Data.xlsx).

Reaction times of the observers to R1, R2 and R3 trials differed significantly in all three experiments. In the main experiment: $F_{2,58}=6.195$, $p<0.004$, Bayes Factor=10, post-hoc comparing R1 and R2, $t_{29}=3.82$, $p<0.001$, $d=0.709$, Bayes Factor=49, R1 and R3, $t_{29}=1.24$, $p=0.226$, $d=0.230$, Bayes Factor=0.4, and R3 and R2, $t_{29}=2.92$, $p=0.006$, $d=0.542$, Bayes Factor=6 (Supplementary

Fig. 1a, top). In the replication experiment: $F_{2,58}$=21.42, $p$<0.001, Bayes Factor=$10^5$, post-hoc comparing R1 and R2, $t_{29}$=3.59, $p$=0.001, $d$=0.667, Bayes Factor=28, R1 and R3, $t_{29}$=2.98, $p$=0.006, $d$=0.554, Bayes Factor=7, and R3 and R2, $t_{29}$=6.95, $p$<0.001, $d$=1.290, Bayes Factor=$1.2*10^5$ (Supplementary Fig. 1b, top). In the control experiment: $F_{2,38}$=33.6, $p$<0.001, Bayes Factor=$1.9*10^6$, post-hoc comparing R1 and R2, $t_{19}$=2.36, $p$=0.029, $d$=0.540, Bayes Factor=2, R1 and R3, $t_{19}$=6.05, $p$<0.001, $d$=1.390, Bayes Factor=2685, and R3 and R2, $t_{19}$=7.13, $p$<0.001, $d$=1.640, Bayes Factor=$2*10^4$ (Supplementary Fig. 1c, top).

Observers' error rates to R1, R2 and R3 trials were also significantly different in all three experiments. In the main experiment: $F_{2,58}$=9.26, $p$<0.001, Bayes Factor=83, post-hoc comparing R1 and R2, $t_{29}$=0.197, $p$=0.845, $d$=0.037, Bayes Factor=0.2, R1 and R3, $t_{29}$=3.95, $p$<0.001, $d$=0.733, Bayes Factor=66, and R3 and R2, $t_{29}$=3.11, $p$<0.001, $d$=0.577, Bayes Factor=9 (Supplementary Fig. 1a, bottom). In the replication experiment: $F_{2,58}$=13.04, $p$<0.001, Bayes Factor=1184, post-hoc comparing R1 and R2, $t_{29}$=2.09, $p$=0.046, $d$=0.388, Bayes Factor=1, R1 and R3, $t_{29}$=4.35, $p$<0.001, $d$=0.808, Bayes Factor=176, and R3 and R2, $t_{29}$=3.32, $p$=0.002, $d$=0.617, Bayes Factor=15 (Supplementary Fig. 1b, bottom). In the control experiment: $F_{2,38}$=3.18, $p$=0.053, Bayes Factor=1, post-hoc comparing R1 and R2, $t_{19}$=3.52, $p$=0.002, $d$=0.808, Bayes Factor=18, R1 and R3, $t_{19}$=0.14, $p$=0.890, $d$=0.0321, Bayes Factor=0.2, and R3 and R2, $t_{19}$=1.99, $p$=0.060, $d$=0.459, Bayes Factor=1, Supplementary Fig. 1c, bottom).

These statistical analyses confirm two expected outcomes. First, observers were faster when the two targets appeared next to each other as opposed to when the targets were on top of each other. This could reflect a bias effect to the horizontal reading direction. Second, observers searched longer when there was only one target letter confirming the effect of observers' extended search for a second target. Observers also made fewer error when there was only one target in the main and the replication experiment but not in the control experiment. This is likely due to the lower base error rate in the control experiment.

More importantly, we also hypothesized that the object- and chunk-based effects in error rates would be large initially, when observers commit many errors in the process of learning the task, and the effect would decrease significantly later, when they reach a good performance with fewer errors. To evaluate this hypothesis, we tested whether observers error rates dropped after the first block, that is whether their performance increased significantly. Indeed, we found that observers made more errors and responded slower in the first block compared to the other blocks. One-way ANOVA of error rates in Experiment 1a showed a main effect of blocks ($F_{3,203}$=31.58, $p$<0.001, Bayes Factor=4.5*$10^{13}$), and post-hoc comparisons of block 1 to block 2-4 confirmed a significant difference ($ts_{29}$>3.38, $p$s<0.002, Bayes Factors>17) (Supplementary Fig. 2a). The same analysis for reaction times also found a main effect of blocks ($F_{3,203}$=94.438, $p$<0.001, Bayes Factor=1.5*$10^{35}$), and a significant post-hoc differences when comparing block 1 to block 2-4 ($ts_{29}$>6.88, $p$s<0.001, Bayes Factors>1.1*$10^{5}$) (Supplementary Fig. 2d).

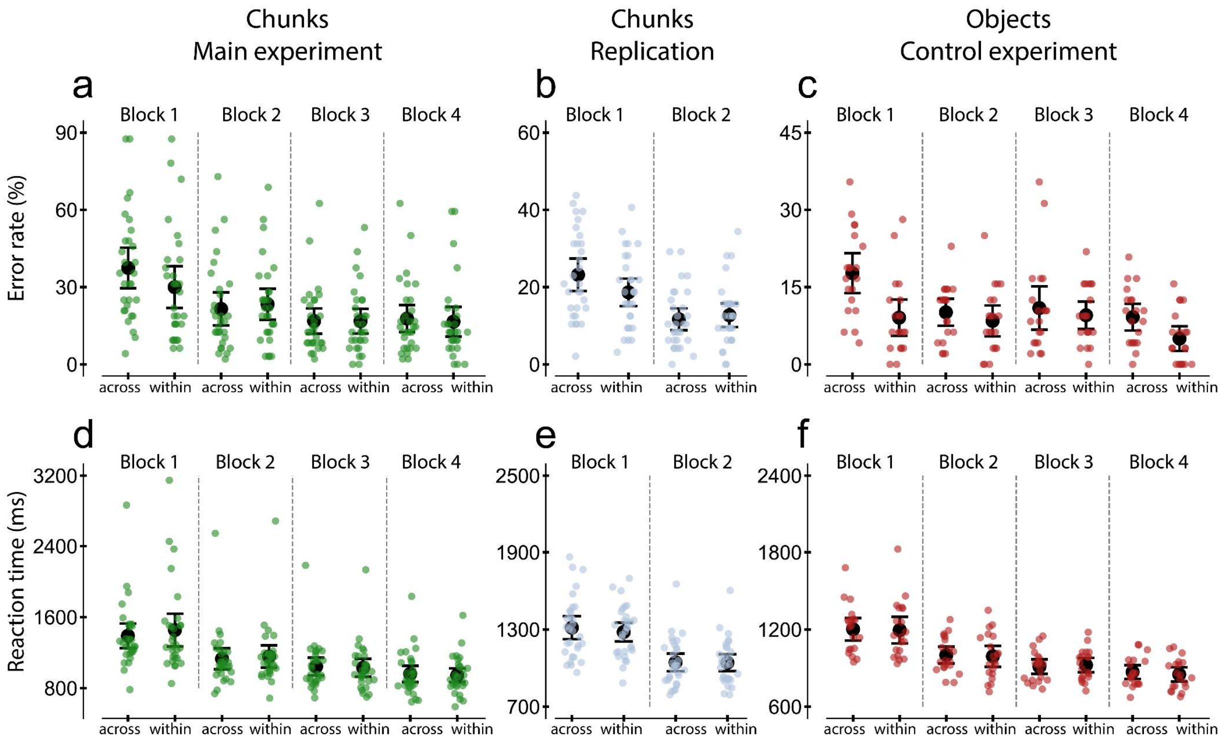


**Supplementary Figure 2**. Mean error rates (**a-c**) and median reaction times (**d-f**) in Experiment 1a (**a, d**), Experiment 1b (**b, e**), and in Experiment 1c (**c, f**) for each block (separated by dotted lines) in the across- and within-chunk/object conditions. On the x axis, the *across condition* denotes trials, in which the two target letters Ts appeared across two chunks/objects, while the *within condition* represents trials, in which the two targets Ts were confined to a single

chunk/object. Dots represent individual observers' performance; error bars indicate the 95% confidence intervals of the mean. Note the different scales on the y axes. n=30 in Exp. 1a (**a, d**), n=30 in Exp. 1b (**b, e**), and n=20 in Exp. 1c (**c, f**). Source data are provided in the Source Data file (Supplementary Figure 2 worksheet tab in Source Data.xlsx).

The analysis of Experiment 1b yielded the same results. Blocks had a main effect on both error rates ($F_{3,203}$=66.57, $p$<0.001, Bayes Factor=$6.9*10^{8}$), with significant advantage of the first block (post-hoc comparing block 1 to block 2, $t_{29}$=7.79, $p$<0.001, Bayes Factor=$9.6*10^{5}$) (Supplementary Fig. 2b), and on reaction times ($F_{3,203}$=184.731, $p$<0.001, Bayes Factor=$1.1*10^{20}$), with a significant disadvantage of block 1 over block 2 ($t_{29}$=10.39, $p$<0.001, Bayes Factor=$3.4*10^{8}$) (Supplementary Fig. 2e).

The control experiment (1c) yielded the same pattern of results as the chunk-based experiments. There was a main effect of blocks both in error rates, ($F_{3,133}$=11.77, $p$<0.001, Bayes Factor=1543), dominated by a significantly larger error in block 1 compared to block 2-4 ($t$s$_{19}$>2.09, $p$s<0.05, Bayes Factors>1), (Supplementary Fig. 2c), and in reaction times ($F_{3,133}$=71.56, $p$<0.001, Bayes Factor=$1.1*10^{25}$), again mostly due to the significantly slower RTs in block 1 comparing to block 2-4 ($t$s$_{19}$>6.24, $p$s<0.001, Bayes Factors>3817) (Supplementary Fig. 2f).

These results clearly support the hypothesis that in every experiment and both in error rates and reaction times, the largest improvement took place between the first and the second block and in subsequent blocks the improvement was negligible. This floor effect diminishing the potential difference between the within- and between-unit effects explains why the chunk/object-based effect in all experiments have disappeared after the first block.

## Experiment 2

### Constructing the catch trials in the familiarity test

For each observer, two *foil pairs*, one horizontal and one vertical, were generated randomly from the *diagonal pairs* the same way as in Experiment 1. We refer to these two foil pairs as *diagonal-pair foils*. More *foil pairs* were created from the *true-pairs* in the following way. Two *horizontal-pair foils* were created by pairing the two top and the two bottom shapes of the two vertical *true pairs*. Two *vertical-pair foils* were created by pairing the two left and the two right shapes of the two horizontal *true-pairs*. We call these four foil pairs *true-pair foils.* Testing the *true-pair foils* against the *true-pairs* contrasts directly the *true-pairs* against those shape combinations that occurred when two *true-pairs* were put together side by side in the search trials creating possible pairs orthogonal to the boundary of the *true-pairs*.

Finally, two *additional foil pairs* were created from the *diagonal-pair foils* in the following way. The top shape of the vertical *diagonal-pair foil* was paired with the left shape of the horizontal *diagonal-pair foil* forming a vertical *foil pair*. The last, horizontal *foil pair* was created by pairing the bottom shape of the vertical with the right shape of the horizontal *diagonal-pair foils*. In the 8 catch trials the 4 *true-pair foils* were tested against these two *additional foil pairs*.

### Comparing average behavior with the chunk- and object-based version of the task

We compared the reaction times (RTs) and error rates (ERs) of the object and chunk version of the paradigm (Supplementary Fig. 3). Observers were faster ($t_{43}$=6.02, $p$<0.001, $d$=0.918, Bayes Factor=$4.6*10^4$) and made fewer error ($t_{43}$=3.84, $p$<0.001, $d$=0.585, Bayes Factor=67) in the paradigm using objects (rectangles). Some part (or all) of this effect could be explained by a generic, maybe attentional based, learning effect because the object version of the paradigm always appeared after all other tasks (the 4-4 blocks of VSL and CBA and the familiarity test). Nevertheless, observers' behavior was very similar across the two stimulus sets.

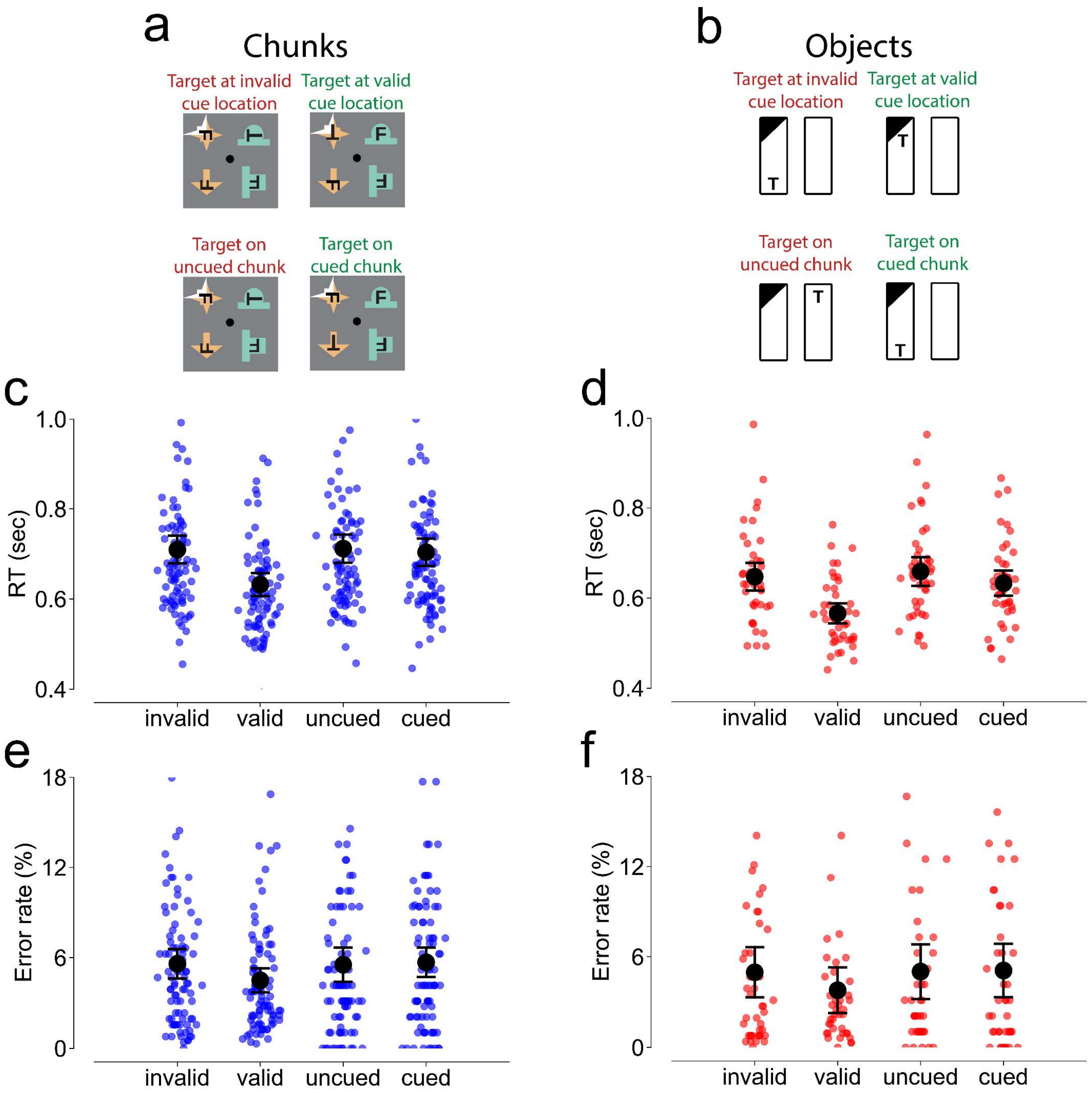


**Supplementary Figure 3**. Median reaction times (**c, d**) and mean error rates (**e, f**) in the four conditions of Experiment 2 using chunks **(a, c, e**) and objects (**b, d, f**). Labels on the x axes: invalid - the target appeared at an uncued location; valid - the target appeared at the cued location; uncued - within the invalid-cue trials, the target appeared on the uncued chunk; cued - within the invalid-cue trials, the target appeared on the cued chunk. Error bars indicate the 95% confidence intervals of the mean; dots represent individual observers' performance. n=90 in the blocks with statistical chunks (**a, c, e** in blue), and n=44 in the blocks with geometric objects (**b, d, f** in red). Source data are provided in the Source Data file (Supplementary Figure 3 worksheet tab in Source Data.xlsx).

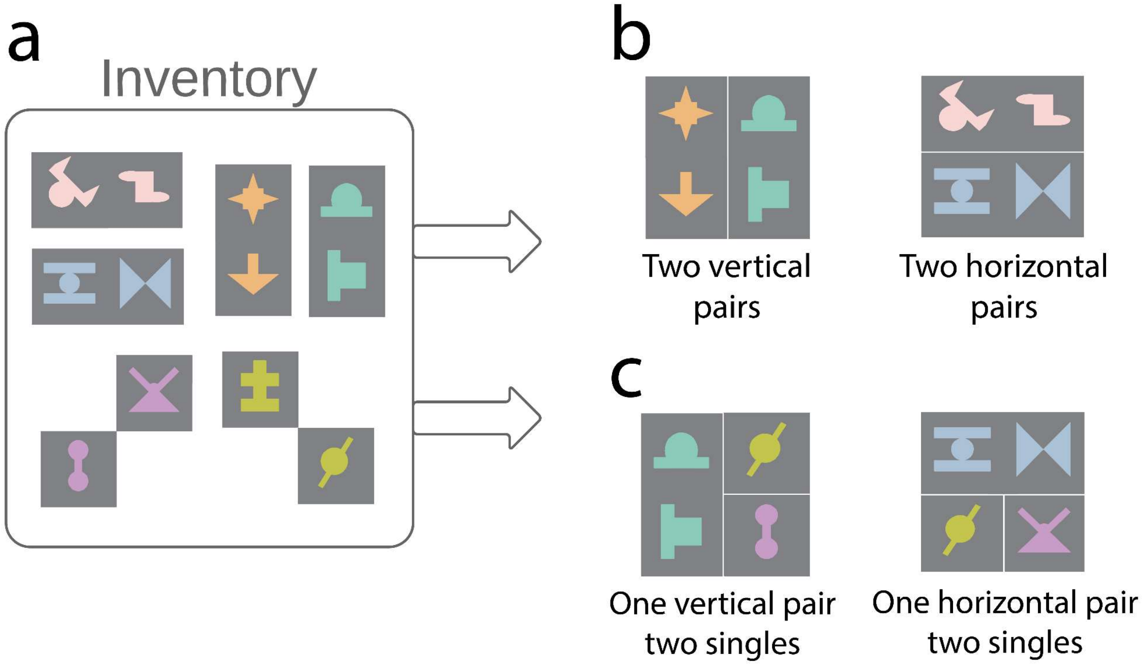


**Supplementary Figure 4**. **a** Shows an example of an inventory from which the true-pairs were generated throughout the experiments. **b, c** Show the four types of trials in the search tasks in all experiments. In this figure, colors are just for demonstration and in the experiments all shapes were black, smaller that in this figure, and were separated by back lines in experiment 1a & b (see Fig. 1b). The separating back lines were removed in experiment 2 (see Fig. 3a). In half of the search trials two true-pairs, either vertically or horizontally oriented, were presented (see two example scenes in **b**). In the other half of the search trials one true-pair, either vertically or horizontally oriented, and two individual shapes from the cross-pairs were presented (see two example scenes in **c**). Note that we do not show here all the unique 2-by-2 scenes for the four trial types that one could generate from the Inventory. However, it is easy to see that only 4 unique 2-by-2 scenes can be generated from the two vertically and two horizontally oriented true-pairs, and 96 unique scenes can be generated from one vertically or horizontally oriented true-pairs and from two individual shapes from the cross-pairs. We presented all 96 unique scenes containing one true-pair and two individual shapes and 12 times the 4 unique scenes containing only two true-pairs. Since in half of the trials containing individual shapes the targets appeared on the individual shape we had the same amount of trials containing two true-pairs and one true-pair and two individual shapes for measuring object-based effects. See Methods, Experiment 1, Visual search paradigm for more details.
