# Supplementary material for: Statistically defined visual chunks engage object-based attention
Source: Nat Commun. 2021 Jan 11;12:272. doi: 10.1038/s41467-020-20589-z (PMC7801661; doi:10.1038/s41467-020-20589-z)
Supplement: Supplementary file 3 — Reporting Summary [file 41467_2020_20589_MOESM3_ESM.pdf]

Corresponding author(s): Gabor Lengyel, Fiser Jozsef

Last updated by author(s): 11/12/2020

Reporting Summary

Nature Research wishes to improve the reproducibility of the work that we publish. This form provides structure for consistency and transparency in reporting. For further information on Nature Research policies, see our Editorial Policies and the Editorial Policy Checklist.

Statistics

For all statistical analyses, confirm that the following items are present in the figure legend, table legend, main text, or Methods section.

n/a	Confirmed	
☐	☒	The exact sample size (*n*) for each experimental group/condition, given as a discrete number and unit of measurement
☐	☒	A statement on whether measurements were taken from distinct samples or whether the same sample was measured repeatedly
☐	☒	The statistical test(s) used AND whether they are one- or two-sided<br>*Only common tests should be described solely by name; describe more complex techniques in the Methods section.*
☐	☒	A description of all covariates tested
☐	☒	A description of any assumptions or corrections, such as tests of normality and adjustment for multiple comparisons
☐	☒	A full description of the statistical parameters including central tendency (e.g. means) or other basic estimates (e.g. regression coefficient) AND variation (e.g. standard deviation) or associated estimates of uncertainty (e.g. confidence intervals)
☐	☒	For null hypothesis testing, the test statistic (e.g. F, t, r) with confidence intervals, effect sizes, degrees of freedom and P value noted<br>*Give P values as exact values whenever suitable.*
☐	☒	For Bayesian analysis, information on the choice of priors and Markov chain Monte Carlo settings
☒	☐	For hierarchical and complex designs, identification of the appropriate level for tests and full reporting of outcomes
☐	☒	Estimates of effect sizes (e.g. Cohen's *d*, Pearson's *r*), indicating how they were calculated

Our web collection on statistics for biologists contains articles on many of the points above.

Software and code

Policy information about availability of computer code

Data collection	We used Matlab (9.0. R2016a) Psychtoolbox (3.0.13) for running the experiments. The data is publicly available at this GitHub repository: https://github.com/GaborLengyel/statistical-chunk-based-attention
Data analysis	We used Python (2.7.15) with numpy (1.15.4), scipy (1.1.0), and matplotlib (3.0.2) libraries to analyze and visualize the results. The code is publicly available at this GitHub repository: https://github.com/GaborLengyel/statistical-chunk-based-attention

For manuscripts utilizing custom algorithms or software that are central to the research but not yet described in published literature, software must be made available to editors and reviewers. We strongly encourage code deposition in a community repository (e.g. GitHub). See the Nature Research guidelines for submitting code & software for further information.

Data

Policy information about availability of data

All manuscripts must include a data availability statement. This statement should provide the following information, where applicable:

- Accession codes, unique identifiers, or web links for publicly available datasets
- A list of figures that have associated raw data
- A description of any restrictions on data availability

The data and code implementing the analyses is publicly available on GitHub: https://github.com/GaborLengyel/statistical-chunk-based-attention

Field-specific reporting

Please select the one below that is the best fit for your research. If you are not sure, read the appropriate sections before making your selection.

☐ Life sciences ☒ Behavioural & social sciences ☐ Ecological, evolutionary & environmental sciences

For a reference copy of the document with all sections, see nature.com/documents/nr-reporting-summary-flat.pdf

Behavioural & social sciences study design

All studies must disclose on these points even when the disclosure is negative.

Study description	Quantitative - experimental
Research sample	Experiment 1: 81 Hungarian university/college students, 53 female, mean age = 21, range = 18-29, 71 right -handed, 49 had normal vision without correction<br>Experiment 2: 98 Hungarian university/college students, 68 female, mean age = 21, range = 18-26, 91 right -handed, 60 had normal vision without correction<br><br>Participants were recruited by a student organization (MADS) that provides job opportunities for Hungarian college students. The samples might only be representative for Hungarian college students. Since, the statistical learning and object-based effects that we investigated here do not depend on the demographic characteristics of the participants, recruiting Hungarian university/college students were the most practical option for our study.
Sampling strategy	Experiment 1: Since we used a novel paradigm in Exp 1. We could not do a statistical power analysis to determine the sample size. Therefore, we aimed to recruit 30-30 participants considering the sample sizes in previous statistical learning (e.g. Fiser & Aslin, 2001, 2005) and object-based processing studies (e.g. Baylis & Driver, 1993; Luck & Vogel, 1997; Vecera, Behrmann, & McGoldrick, 2000, Egly et al., 1994; Vecera, 1994).<br>Experiment2: We estimated the effect size of the original object-based attention (OBA) reported in previous studies (Egly et al., 1994; Lee et al., 2012; Moore et al., 1998; Shomstein & Yantis, 2004; Vecera, 1994) and found that, on average, OBA has a small effect size (Cohen's d = 0.22). Since chunk-based attention (CBA) is likely to be even weaker than OBA, we assumed that CBA would yield an effect half as strong as in OBA. Asking for 60% probability to find the CBA, we established that our study required a sample size of 104 observers. We aimed at one hundred observers and managed to recruit 98 university students.
Data collection	Each participant sat in front of a monitor in a dimly lit room. The experimenter first explained the task to the participant, then left the room and the participant were left alone in the room during the experiment. The experimenter were sitting outside in front of the room in case the participant needed something (e.g. further clarification regarding the task). The stimuli appeared on the display and the participant had to push keys on a keyboard to give response to the stimuli. The experiment followed a within subject design with experimental conditions randomly shuffled across the trials of the experiment, therefore the experimenters were completely blind to the experimental conditions.
Timing	Experiment 1 a,b and Experiment 2 was conducted in 2017 March-June. Experiment 1 c (control) was conducted in 2019 May.
Data exclusions	We excluded one participant from Experiment 1b who explicitly noticed the statistical structure of the pairs/chunks. As in Experiment 1, observers with explicit knowledge of the chunks were excluded (5/98) from Experiment 2. We excluded three additional observers from Experiment 2 because they did not finish the experiment, thus data of 90/98 observers were analyzed in Experiment 2.
Non-participation	There were three participants who did not read carefully the description of experiment 2 and thought the experiment would only take one hour (usually our experiments take an hour). After an hour, these participants said they would need to leave and they left without finishing the experiment.
Randomization	Participants were randomly allocated into the experimental groups. All experiments were with within-subject design and the experimental conditions were randomly shuffled across the trials in each experiment.

Reporting for specific materials, systems and methods

We require information from authors about some types of materials, experimental systems and methods used in many studies. Here, indicate whether each material, system or method listed is relevant to your study. If you are not sure if a list item applies to your research, read the appropriate section before selecting a response.

Materials & experimental systems

n/a	Involved in the study
☒	☐ Antibodies
☒	☐ Eukaryotic cell lines
☒	☐ Palaeontology and archaeology
☒	☐ Animals and other organisms
☐	☒ Human research participants
☒	☐ Clinical data
☒	☐ Dual use research of concern

Methods

n/a	Involved in the study
☒	☐ ChIP-seq
☒	☐ Flow cytometry
☒	☐ MRI-based neuroimaging

Human research participants

Policy information about studies involving human research participants

Population characteristics	See above
Recruitment	Participants were recruited by a student organization (MADS) that provides job opportunities for Hungarian college students. Self-selection bias could not influence our results because 1) the participants were entirely naive to the experiment, 2) experimental conditions were randomly shuffled in a within-subject design, and 3) the statistical learning and object-based effects that we investigated here do not depend on the demographic characteristics of the participants.
Ethics oversight	The experimental protocols were approved by the Ethics Committee for Hungarian Psychological Research

Note that full information on the approval of the study protocol must also be provided in the manuscript.
